# Camouflage in lichen moths: Field predation experiments and avian vision modelling demonstrate the importance of wing pattern elements and background for survival

**DOI:** 10.1111/1365-2656.13817

**Published:** 2022-10-08

**Authors:** Cassandra J. Mark, James C. O'Hanlon, Gregory I. Holwell

**Affiliations:** ^1^ School of Biological Sciences The University of Auckland Auckland New Zealand; ^2^ School of Science and Technology University of New England Armidale New South Wales Australia

**Keywords:** artificial models, background matching, camouflage, colour analysis, crypsis, lichen resemblance, moth

## Abstract

1. Background matching is perhaps the most ubiquitous form of defensive camouflage in the animal kingdom, an adaptive strategy that relies on the visual resemblance between a prey organism and its background to promote concealment from predators. The importance of background matching has been acknowledged for over a century, yet despite its renown and apparent pervasiveness, few studies exist that have objectively quantified its occurrence and tested the functional significance of background matching in a specific animal study system.
2. The North Island lichen moth *Declana atronivea* presents a fascinating system to investigate such anti‐predator coloration. This species possesses high contrast black and white forewings that appear to resemble lichen. Here we assessed the contribution of background matching to the antipredator defence of *D. atronivea* using field predation experiments with realistic models.
3. We found that *D. atronivea* coloration confers a significant survival advantage against native avian predators when on lichen backgrounds compared to bark backgrounds, with an intermediate level of predation occurring when models were near, but not on lichen. This suggests that *D. atronivea* wing patterns are an adaptation for background matching.
4. We subsequently used calibrated digital photography, avian vision modelling and image analysis techniques to objectively quantify the degree of background matching exhibited by *D. atronivea* and assessed the contribution of different visual elements (colour, luminance and pattern) to camouflage in this species. Only the pattern elements of *D. atronivea* presented a close match to that of the lichen backgrounds, with both chromatic and achromatic cues found to be poor predictors of background matching in this species.
5. This study is one of the first to integrate vision modelling, quantitative image analysis and field predation experiments using realistic models to objectively quantify the level and functional significance of background matching in a real species, and presents an ideal system for further investigating the interrelation between multiple mechanisms of camouflage.

## Graphical abstract

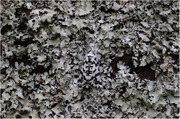

## 1 INTRODUCTION

The most common conception of camouflage is that of an animal blending into its surroundings; a visual harmony between prey and background that promotes concealment from predators. This principal of camouflage has previously been called ‘general resemblance’ (Wallace, 1889), ‘background picturing’ (Thayer, 1909), ‘cryptic resemblance’ (Cott, 1940) and ‘crypsis’ (as adopted from Endler's, 1978 definition of crypsis), although is now more commonly referred to as ‘background matching’ (Endler, 1978; Merilaita & Stevens, 2011). More specifically, background matching defines a camouflage strategy in which the appearance of an animal matches the visual components (colour, luminance, pattern and texture) of one or more backgrounds such that it becomes visually indistinct from its surroundings, allowing it to blend in and remain undetected by predators (Cuthill, 2019; Merilaita & Stevens, 2011).

The idea of background matching has been acknowledged for over two centuries (i.e. Darwin, 1809; Poulton, 1890; Wallace, 1889), and was a fundamental phenomenon for early demonstrations of the process and function of adaptation (Merilaita & Stevens, 2011). While these historical works, and those by contemporaries (i.e. Cott, 1940; Thayer, 1896, 1909), have been invaluable for providing a foundation of camouflage theory, they presented largely subjective accounts of the function of animal coloration based on human perception. It was not until the latter part of the 20th century that scientists gained a more thorough understanding of animal visual systems, and with it, the recognition that animals see the world differently from us and each other (Caro et al., 2017). Analysing animal coloration through the visual systems of relevant receivers is thus imperative because it is the intended viewer's perspective that has created the selection pressures leading to the evolution of such coloration.

To quantify how a particular camouflage trait promotes concealment from predators, it is first necessary to understand the properties of the relevant predators' visual systems. The visual sense of animals varies considerably among taxa (Troscianko et al., 2017); variation in eye size and shape, the number of visual pigments, the type and number of photoreceptors, as well as retinal and post‐retinal processing, all contribute to the unique visual systems found across the animal kingdom (van den Berg et al., 2020).

Photoreceptor class, density and sensitivity are particularly important for chromatic (colour) and achromatic (luminance) visual processing, as well as the perception of spatial information (patterns). Chromatic perception (hue and saturation) enables the detection of spectral information, that is, differences in light spectra (independent of intensity) owing to the stimulation of at least two different photoreceptors with divergent spectral sensitivities (Osorio & Vorobyev, 2005). Colour vision therefore permits the discrimination of objects and surfaces based on their chromatic aspect (Osorio & Vorobyev, 2005). More specifically, it is thought that colour vision is optimally tuned to the detection of larger objects, as well as short‐distance recognition (Jones & Osorio, 2004; Olsson et al., 2018; Osorio & Vorobyev, 2005). Conversely, luminance (light intensity) vision enables the perception of brightness and lightness, and is thought to be important for the detection of more detailed spatial information, such as motion, shape, texture and pattern, and may mediate long‐distance recognition (Osorio, Miklósi, et al., 1999; Osorio & Vorobyev, 2005; Osorio, Vorobyev, et al., 1999). Chromatic and achromatic signals are therefore likely to be used in separate visual tasks, although the relative importance of each to background matching is unclear. Some studies have suggested that luminance may be a more critical cue than colour in terms of background matching since, as mentioned above, it is assumed that predators rely on achromatic contrast to discriminate small objects and resolve finer spatial details, such as patterns (Jones & Osorio, 2004; Kang et al., 2016; Osorio, Miklósi, et al., 1999; Stuart‐Fox et al., 2006). Other studies, however, indicate that both chromatic and achromatic information are equally important, and the combination of these cues aids in visual discrimination (e.g. Stobbe et al., 2009). Perhaps the relative importance of these visual characteristics for background matching differs between species and ecological context (Stuart‐Fox et al., 2006).

The functional significance of background matching has been demonstrated in both natural and artificial systems (e.g. Bond & Kamil, 2002; Duarte et al., 2018; Merilaita & Dimitrova, 2014; Michalis et al., 2017; Price et al., 2019; Stevens et al., 2015; Xiao & Cuthill, 2016). The most famous example of background matching is perhaps the peppered moth *Biston betularia*. This species exists as two different coloured morphs: *typica*, with pale wing coloration, and *carbonaria*, the dark melanic form. The adaptive significance of this dimorphism in relation to background matching was first demonstrated in studies by Kettlewell (1955, 1956). He noticed that *typica* had a selective advantage in unpolluted woodland areas where lichen‐covered trees were common and effective substrates for concealment, while *carbonaria* had greater survival in polluted areas where lichen was replaced with soot particulates (Kettlewell, 1955, 1956). Evidence of the adaptive value of this camouflage system was more recently presented by Walton and Stevens (2018), who further quantified the level of background matching of *B. betularia* against its different substrates through avian vision models and image analysis, and related this directly to survival against avian predators in controlled field experiments with realistic models. The adaptive value of background matching has also been demonstrated in *Peromyscus polionotus* mice which show colour polymorphism across different geographic locations; inland populations have a dark‐brown or fawn pelage that were shown to provide better concealment against the agricultural fields they inhabit, while the coastal populations have a much paler pelage which offer greater background matching against their sand dune habitats (Nokelainen et al., 2020; Vignieri et al., 2010).

**FIGURE 1:**
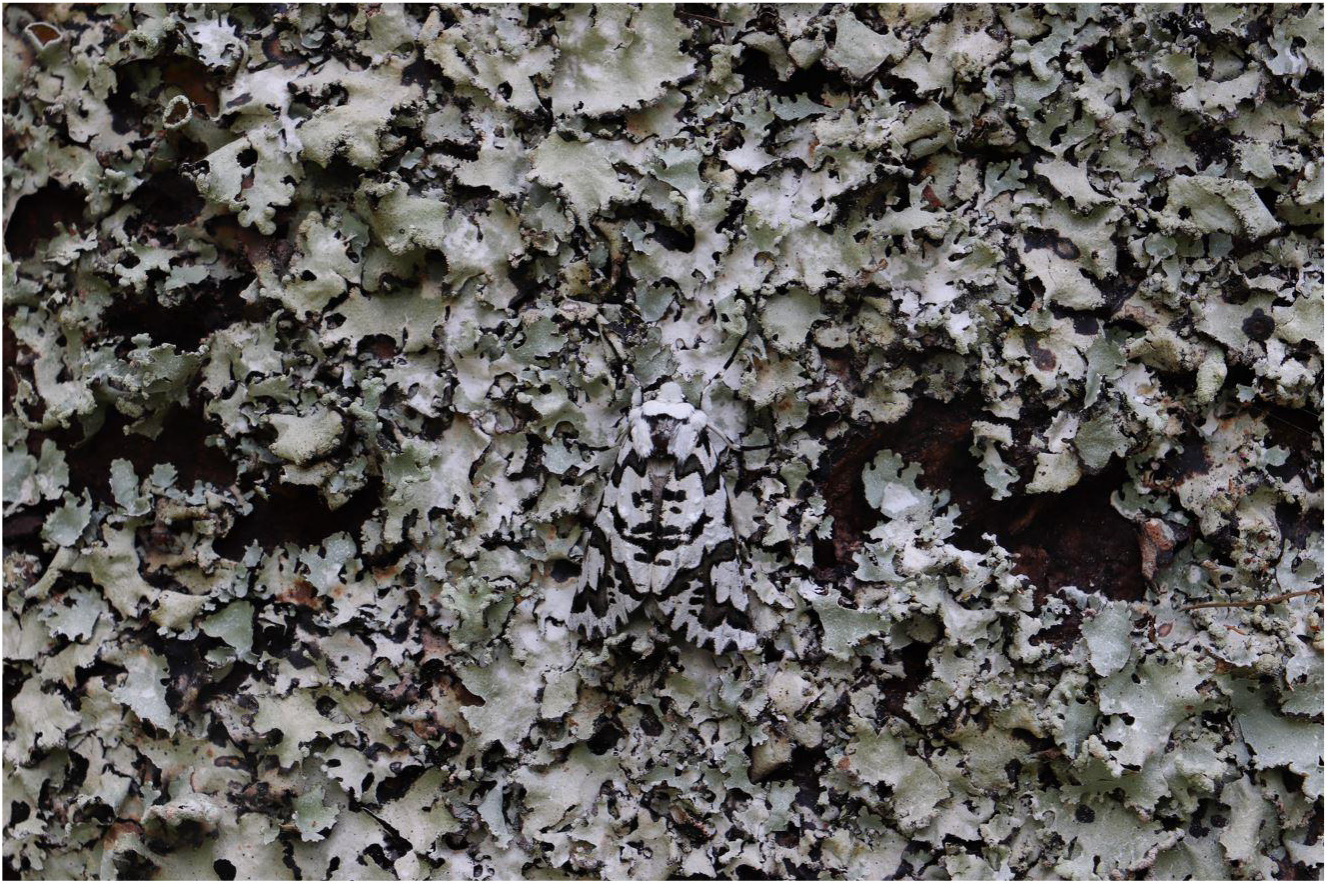
*Declana atronivea* moths have intricate black and white forewing patterns that appear to resemble lichen and may have an adaptive function for camouflage through background matching.

The North Island lichen moth *Declana atronivea* (Walker, 1865), an endemic geometrid moth from Aotearoa | New Zealand, is so named because the intricate black and white forewing patterns bear a striking resemblance to lichen (Figure 1). This resemblance is such that, to the untrained human eye, *D. atronivea* resting on lichen is virtually indistinguishable from its background substrate (pers. obs.; Figure 1). Such an appearance suggests a possible background matching function in this species.

Here we assessed the contribution of background matching to the antipredator defence of *D. atronivea* in the field. To do so, we employed field predation experiments using moth models based on the actual colour patterns of *D. atronivea* as calibrated through spectrophotometry. Specifically, we tested whether the appearance of *D. atronivea* reduces predation when (a) resting on a background of lichen, and (b) when in close proximity to lichen, relative to (c) when resting on bark substrates devoid of lichen. It was predicted that if the colour patterns of *D. atronivea* function to provide concealment through background matching, then models placed on lichen should be attacked less often than the other treatments. The second treatment (b) was included to determine if proximity to lichen alone provided a survival advantage. Proximity to lichen alone might reduce detection by predators if a broad background of bark and lichen resulted in increased background complexity (Dimitrova & Merilaita, 2010, 2012; Merilaita, 2003). Additionally, with patches of lichen nearby, moths may be more likely to be misclassified by predators as pieces of lichen, gaining the benefits of element‐imitation masquerade (Skelhorn, 2015; Skelhorn et al., 2010; Skelhorn & Ruxton, 2010).

A further aim for this study was to objectively quantify the occurrence and degree of background matching in *D. atronivea* using calibrated digital photography, receiver vision models and quantitative image analysis techniques. By measuring the colour, luminance and pattern properties of the moths in conjunction with different background substrates as modelled under blue tit *Cyanistes caeruleus* vision, we were able to estimate how *D. atronivea* appear against natural backgrounds to an avian visual system. It was predicted that if the coloration of *D. atronivea* has an adaptive function for background matching on lichen, then the colour, luminance and pattern contrasts between moth and background should be lowest on the lichen substrates, thus providing greater background matching as compared to bark backgrounds.

This research is, to our knowledge, one of only two studies that integrate vision modelling, image analysis techniques and predation experiments using realistic models to objectively quantify the level and functional significance of background matching coloration in a real moth species (Walton & Stevens, 2018). This integrated approach allows us to assess which aspects of an animal's appearance (colour, luminance and/or pattern) contribute most to observed patterns of predation.

## 2 MATERIALS AND METHODS

### 2.1 Field survival experiments with artificial models

Field predation experiments were carried out using paper moth models to examine the potential survival benefit of background matching in *D. atronivea*. The artificial models represented a natural *D. atronivea* specimen and were placed on three different backgrounds: lichen, bark and near lichen (i.e. on bark but in close proximity to a lichen patch). Lichen patches consisted of various assemblages that primarily included *Parmelia*, *Parmotrema*, *Flavoparmelia* and *Hypotrachyna* species.

### 2.2 Creating the models

The artificial models were produced from a photograph of a single fresh, intact specimen using Adobe Photoshop (ver. 22.4.2). To ensure that the models were appropriately calibrated to bird vision and reflected as close to real *D. atronivea* as possible (Merrill et al., 2012), we first used spectrophotometry to quantify the wing coloration of a sample of moths (*n* = 10). We also obtained reflectance measurements of different printing materials (paper and ink) and used the ‘r’ package, ‘pavo’ (Maia et al., 2019), to compare these spectral data with that of *D. atronivea* forewing colour patches to find the closest colour match (see Supplementary Material for more detailed methodology). The colour patterns of the models were based on a single specimen to control for variation in pattern elements among individuals and to ensure that any differences in field survival of models were explained by differences in background alone. The models were scaled to accurately match the size of real *D. atronivea* specimens (22 mm resting wingspan) and then printed at 1200 dpi using a RICOH MP C4504 colour laser printer onto Whatman filter paper (No.1 12.5 cm) that had been glued to standard white A4 card. The models were made waterproof by coating them with Plasti‐Kote Clear Acrylic spray paint. Moth bodies were made using pastry: an edible, non‐toxic mixture of lard and plain flour in a 1:3 ratio. They were rolled into small cylinders approximately 15 mm long and left to freeze overnight. Once hardened, a small pin was placed through the bodies and then glued to the paper models using non‐toxic PVA glue. The moth bodies were small enough that they were only just visible beneath the model so as to not present a salient target that may inadvertently attract avian predators, while still providing space for attack marks to be observed.

### 2.3 Field survival experiments

The experiment was conducted at two locations in West Auckland: Oratia Reserve (owned by the University of Auckland) (36°55′01.3″S 174°36′11.5″E), which is a podocarp, broadleaf and kauri forest; and Matuku Reserve (36°51′40.3″S 174°28′55.8″E), which is predominately a lowland broadleaf and kanuka *Kunzea ericoides* forest with ridges of emergent tanekaha *Phyllocladus trichomanoides* and kauri *Agathis australis*. These sites were selected for their native forest habitats, populations of native insectivorous birds (tui *Prosthemadera novaeseelandiae*, pīwakawaka (New Zealand fantail) *Rhipidura fuliginosa* and miromiro (North Island tomtit) *Petroica macrocephala*), and abundance of foliose lichen.

**FIGURE 2:**
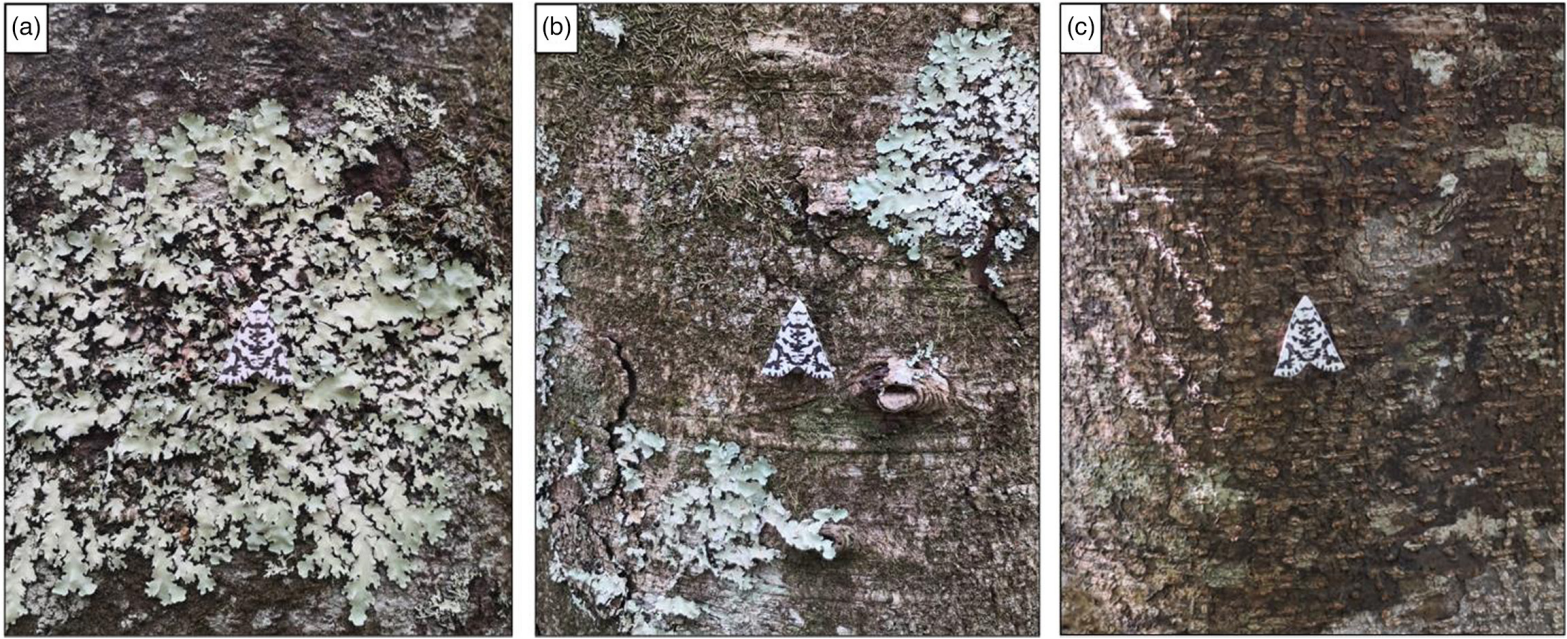
Placement of the artificial moth models for the (a) lichen treatment, (b) off‐lichen treatment and (c) bark treatment.

The survival data for this experiment were collected in January 2021 to coincide with the active season of *D. atronivea*. The experiments at each site were carried out over two separate weeks. Two sampling blocks were established at each location, running approximately 100 m long and 40 m wide. A sample of 150 models were placed over the two blocks at each site, with 25 replicates pinned on each substrate type (lichen patches, ‘off‐lichen’ patches or plain bark) per block (total sample size of 300 models; 100 replicates per substrate type across both sites), placed on individual tree trunks that were at least 5 m apart. Patches of foliose lichen measuring at least 5 × 5 cm in area were selected for the lichen treatment to ensure that the models were encompassed within the substrate (Figure 2a). For the off‐lichen treatment, the models were placed on bark but approximately 5 cm away from a lichen patch (Figure 2b). The criteria for the plain bark treatment were that the substrate only consisted of bark and the tree was devoid of any moss or lichen elements (Figure 2c). The placement of the models on the trees was randomised so that they were pinned at different heights (between 0.3 and 2.5 m) and compass directions. This was to standardise for the irregular occurrence and positioning of foliose lichen on the trees. Care was taken to ensure all models were pinned in the same orientation, at 0° of the vertical plane. Models were chosen at random when pinning to avoid any selection bias and were designated an individual ID (substrate type and replicate number) for ease of monitoring. Pinning height, compass direction and tree type were also recorded. Models were checked at three different time intervals (24, 48 and 72 h) for evidence of predation. Any clear indication of avian attack (i.e. visible beak marks, part or all of the body missing or wings partly or entirely removed) was recorded. For statistical analysis, the results were subsequently censored as 1 for ‘attacked’ and as 0 if the models ‘survived’ until the end of the experiments (at 72 h) or had other signs of attack or damage (such as from ants or snails).

### 2.4 Survival analyses

The predation data were analysed in ‘r’ using a Cox proportional hazards regression model (‘*coxph*’ function in the ‘survival’ package; Therneau, 2021). This analysis allowed us to compare the ‘hazards’ or ‘risks’ of different treatment groups to a particular event, which in this case was the predation risk (or risk of attack) over time for the models placed on lichen, off‐lichen or bark substrates. The time to attack (recorded as 24, 48 or 72 h) and the risk of attack (with data censored either 1 or 0 as described above) were the response variables, with treatment (background type), block number (1–4) and location (Oratia or Matuku) as the predictor variables. The resulting Wald statistic was used to assess statistical significance. The exponentiated coefficients of the covariates explained the hazard ratios (or effect sizes), and the regression coefficients denoted whether the hazard (risk of attack) was higher (positive value) or lower (negative value) for each variable. Pairwise comparisons using the log‐rank test were computed using the ‘pairwise_survdiff’ function of the ‘survival’ package. Survival estimates of the different treatments were derived from the ‘survfit’ function and subsequently used to produce a plot of survival curves.

### 2.5 Colour analyses

Calibrated digital photography and image analysis techniques were used to objectively quantify the level of background matching in *D. atronivea* moths. By measuring the colour, luminance and pattern properties of the moths in conjunction with different background substrates as modelled under avian vision, we were able to estimate how *D. atronivea* appear against natural backgrounds to an ecologically relevant visual system. This provided a perspective for understanding the function of the coloration in this species.

### 2.6 Image acquisition

Digital photographs of dead (but intact) *D. atronivea* moths (*n* = 11) were taken on the bark of native trees (kohekohe *Dysoxylum spectabile*; kowhai *Sophora microphylla*; puriri *Vitex lucens*; taraire *Beilschmiedia taraire*; titoki *Alectryon excelsus*; totara *Podocarpus totara*) and on lichen substrates that were found on kauri (‘kauri‐lichen’, which primarily consisted of a mixture of *Parmotrema*, *Hypotrachyna*, *Heterodermia* lichen species) and titoki (‘titoki‐lichen’, including lichen species of the *Parmelia*, *Flavoparmelia*, and *Punctelia* genera) located in the grounds of the University of Auckland (between 36°50′59.0″S 174°46′10.2″E and 36°51′00.0″S, 174°46′16.0″E) during January 2020. These substrates were selected because they were found to be common in the forests of Oratia and Matuku reserves where the field predation experiments took place.

The moth specimens were each mounted onto pins and then pinned to the substrate during image acquisition. All moth specimens were photographed on the same individual tree of each tree species. Care was taken to ensure the specimens were all pinned at the same orientation (0° of the vertical plane), although pinning height and placement around the tree trunk was randomised so that the images did not represent the same patch of background. All photos were taken at a distance of 0.5 m from the tree. A white Spectralon standard (99% reflectance; Labsphere) attached to a scale bar was placed at the side of each photo. Photographs were captured during the day and under cloudy conditions to ensure more even and diffuse lighting. Acquisition of the digital images was carried out following the protocols described in Troscianko and Stevens (2015) and the corresponding Image Calibration and Analysis Toolbox user guide (see Supplementary Material for more detailed methodology).

### 2.7 Image analyses

Visual modelling and analysis of the images were conducted using the open‐source Multispectral Image Calibration and Analysis (MICA) toolbox (Troscianko & Stevens, 2015) and the integrated Quantitative Colour Pattern Analysis (QCPA) Framework (van den Berg et al., 2020) for ImageJ (version 1.5.3; Schneider et al., 2012) (see Supplementary Material for more detailed methodology).

#### 2.7.1 Background matching: Colour and luminance discrimination

We used a log form of the receptor noise‐based visual discrimination model (RNL) to quantify the level of chromatic distinguishability between the moths and different background substrates under the blue tit *C. caeruleus* visual system (Vorobyev & Osorio, 1998), and a modified achromatic version of the model to assess luminance (Siddiqi et al., 2004) following the conventions of previous studies (Price et al., 2019; Robledo‐Ospina et al., 2017; Stevens et al., 2015, 2017). The model predicts the perception of contrast between the regions of interest (ROI; the moth and its background substrate) based on the respective signal to noise ratios of each channel under the blue tit visual system by reconstructing the colour distances (Δ*S*) between the two spectra (Price et al., 2019; Troscianko & Stevens, 2015). The output is a calculation of ‘just noticeable difference’ (JND), where values less than one are considered indiscriminable while values above one suggest an increasing probability of discrimination and thus indicate a reduction in the level of background matching (Siddiqi et al., 2004; Troscianko & Stevens, 2015). However, it should be noted that the use of the RNL model for achromatic discrimination lacks strong behavioural validation and thus the luminance JND values should be interpreted with caution (Duarte et al., 2018).

To account for the spatial resolution of the receiver (blue tit), cone catch images were first run through Gaussian Acuity Control using a spatial acuity value of six cycles per degree (CPD; as suggested within the QCPA toolbox) and rescaled to 5 px per minimum resolvable angle (MRA). This was done for the whole image (i.e. not the separate ROIs) and was run separately for viewing distances of 500 and 1000 mm (0.5 and 1 m). Acuity control effectively removes any information that would not be available to blue tits viewing a scene at each set distance.

Colour and luminance measurements of each ROI were then run on the acuity‐controlled images, with luminance based on the blue tit double cone channel. JND values were subsequently calculated for the chromatic and achromatic contrasts between the moths and backgrounds across the two viewing distances. The chromatic and achromatic JND values were analysed as separate metrics in subsequent statistical analyses.

#### 2.7.2 Background matching: Pattern

Background pattern matching was quantified via a similar protocol to that described above, although an additional granularity analysis was performed. This type of analysis is based on early‐stage spatial filtering and low‐level neuro‐physiological image processing (Troscianko & Stevens, 2015) and has been widely used for measuring animal markings, such as in cuckoo‐host eggs (Spottiswoode & Stevens, 2010; Stoddard et al., 2019), fish (Tyrie et al., 2015; Watson et al., 2014) and cephalopods (Hanlon et al., 2009). During granularity analysis, Fast Fourier Transform bandpass filtering is applied to each image, resulting in the image being filtered across a ‘granularity spectrum’ of multiple spatial frequency scales. The pattern ‘energy’ of the image is measured at each scale as the standard deviation of the pixel values (Price et al., 2019). The measurement scale used here was a step multiplier of two, beginning with two pixels and ending at 1200 pixels. The resulting data were used to directly compare the pattern energy difference between the moth and background ROIs using the ‘pattern energy difference’ (PED) calculator within the MICA toolbox, giving a measure of background pattern matching. Pattern energy difference is a value of absolute difference between the pattern spectra of the two ROIs across all spatial scales. Low values indicate similarity in pattern energy between the moths and respective backgrounds, suggesting a greater level of background pattern matching than higher values. Overall, a PED value for each moth on each background at each of the viewing distances was derived from the granularity analysis.

### 2.8 Statistical analysis

Colour JND, luminance JND and PED data were all separately analysed in ‘r’ (ver. 4.1.0; R Core Team, 2021) using a linear mixed‐effects model (lmer function of the lme4 package (ver. 1.1‐27.1; Bates et al., 2015) and the lmerTest package extension for obtaining *p*‐values (ver. 3.1‐3; Kuznetsova et al., 2017)). This model was chosen to account for the repeated measurements on each individual moth as a random effect, and using background and viewing distance fixed effects. For each of the camouflage metrics, the model was performed in a stepwise manner to determine which fixed effects or combination of fixed effects presented the greatest predictive power for that model. Once the parameters for the models were determined, post‐hoc tests for multiple comparisons were conducted using the emmeans function of the emmeans package (ver. 1.6.2‐1; Lenth, 2021). This function obtained the estimated marginal means (or least‐squares means) of the model factors, allowing for pairwise comparisons between the levels of the fixed effects.

### 2.9 Ethics statement

Licences and permits to conduct the field research at Oratia and Matuku reserves were not required; Oratia Reserve is owned by The University of Auckland and Matuku Reserve does not require formal consent, although permission to access the site was kindly granted by John Staniland from Forest and Bird. Approval for the use of moth models for the field predation experiments was granted by the Auckland Animal Ethics Committee (AEC) (REF‐002153). Permission to collect *Declana atronivea* at Tongariro National Park and Pureora Forest Park was granted by the Department of Conservation (Te Papa Atawhai) and local iwi, Ngāti Tuwharetoa (permit number 67979‐RES).

## 3 RESULTS

### 3.1 Predation experiments

**FIGURE 3:**
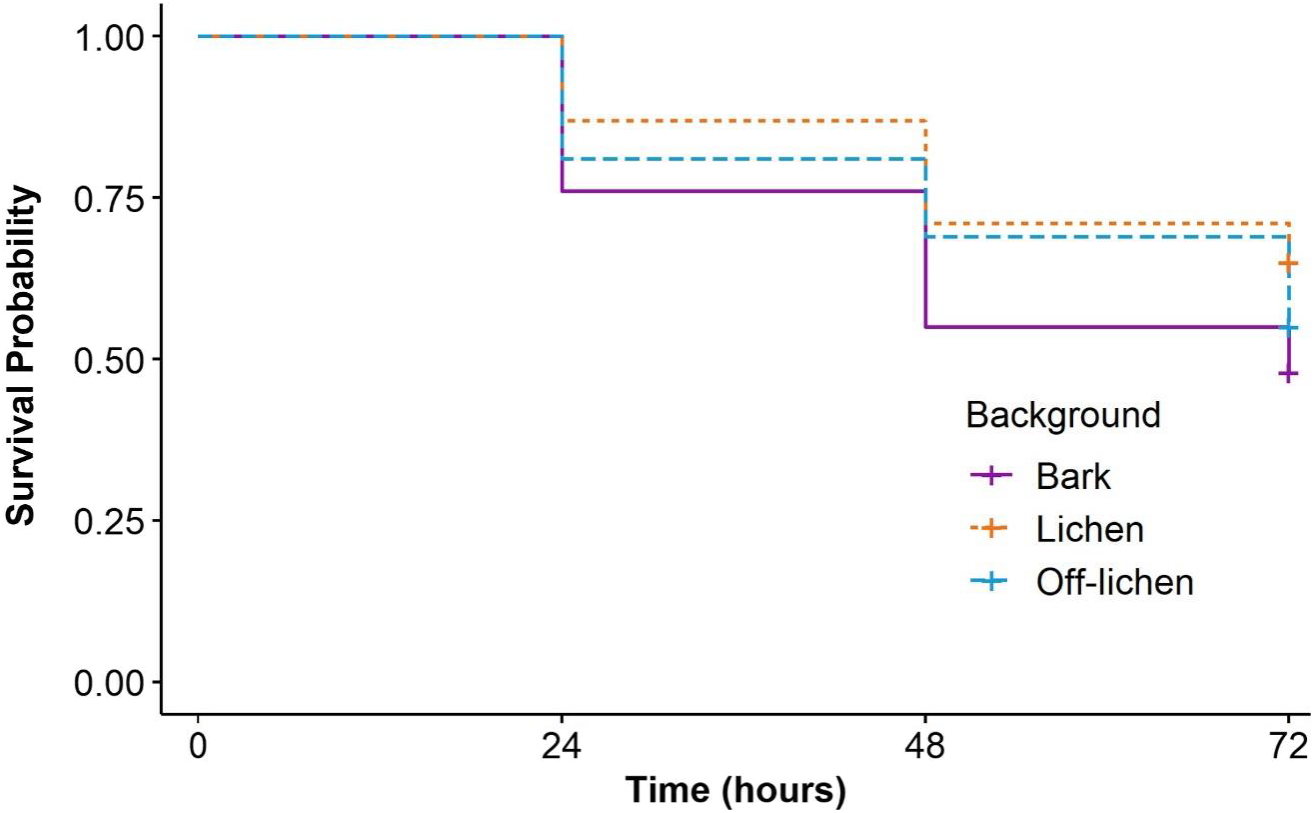
Survival curves of moth models on the bark (solid purple line), lichen (dashed orange line) and off‐lichen (dashed blue line) backgrounds. The curves represent the probability of surviving predation by avian predators as a function of time and are based on Kaplan–Meier survival estimates that account for censoring due to survival to the end of the study. Overall, models on bark had significantly lower survival (0.48 ± 0.05) compared to those on lichen (0.65 ± 0.04) (*p* = 0.01) at the end of the 72‐h period, whereas the survival rate of off‐lichen (0.55 ± 0.05) did not differ significantly from bark (*p* = 0.23) nor lichen (*p* = 0.18).

Survival rates of the moth models were measured over 72 h across the two study sites and were found to differ across the treatments: of the 100 model replicates per substrate type, 52 were attacked on bark, 45 were attacked on off‐lichen and 35 were attacked on lichen. The initial Cox proportional hazards model revealed no significant interaction between location, block and background substrate (all *p*‐values > 0.1), so the interaction term was removed from the model. A second model with all terms as main effects showed that location and block were not significant (location *p* = 0.11; block *p* = 0.10) so they were also removed from the model. The final Cox proportional hazards model was run with only background treatment as the main effect, which was found to be statistically significant (overall model Wald test = 6.31, df = 2, *p* = 0.04). Models placed on the bark backgrounds had a significantly lower survival probability compared to those on lichen (*p* = 0.01) (Figure 3). Overall, the regression coefficients of the model indicate that both lichen (coef = −0.5487) and off‐lichen (coef = −0.2448) presented a lower risk of predation compared to those on bark. The effect size or hazard ratio, as determined by the exponential coefficients, show that lichen backgrounds reduced the risk of predation by a factor of 0.57, or 43%, compared to bark, while the off‐lichen backgrounds reduced the hazard by only 0.78, or 22%. There was, however, no overall significant difference in survival probability between off‐lichen and bark (*p* = 0.23), or off‐lichen and lichen (*p* = 0.18).

### 3.2 Colour analysis

#### 3.2.1 Chromatic matching

**FIGURE 4:**
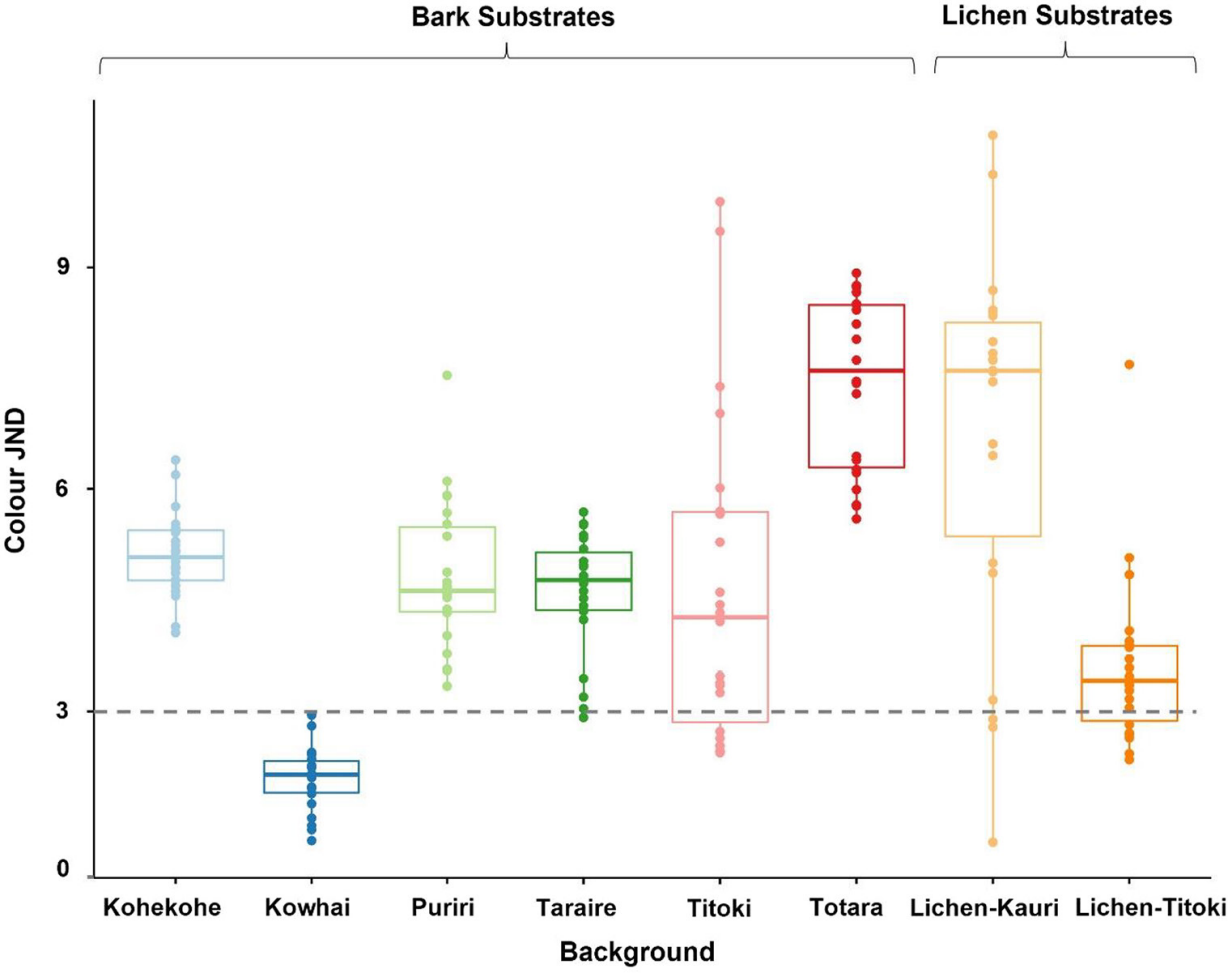
Chromatic (colour) discrimination values (JNDs) for moths on the different background substrates as modelled under blue tit vision. Moths placed on kowhai had the lowest JND values (mean JND = 2.09 ± 0.09), suggesting that they would be difficult to distinguish from the background under the current avian vision model. All other substrates presented poor colour matches to the moths, particularly totara (mean JND = 7.44 ± 0.25) and lichen‐kauri (mean JND = 6.79 ± 0.53). Boxplots show median values (middle line), interquartile range (box) and the range values including some outliers (dots which extend beyond the min and max of the boxplot). The horizontal grey dashed line indicates JND = 3 for comparison.

The level of chromatic matching, as based on the colour JND values, was significantly predicted by background type (*F*_7_ = 32.32, *p* < 0.001), but there was no effect of viewing distance (*F*_1_ = 0.62, *p* = 0.43) nor any significant interaction between background type and viewing distance (*F*_7_ = 0.12, *p* = 0.99) (Figure 4). The mean JND values of moths placed on kowhai bark was found to be significantly different to those on all other background types (all *p*‐values < 0.005), and interestingly, kowhai bark was the closest chromatic match to the moths (mean JND ± SE = 2.09 ± 0.09). Such low chromatic differences suggest that moths resting on this background substrate may not be easily distinguishable to avian predators under the current vision model and viewing conditions. Lichen‐titoki was the next closest chromatic match (mean JND = 3.63 ± 0.245) and was significantly different to kohekohe (*p* = 0.009), kowhai (*p* = 0.006), lichen‐kauri (*p <* 0.001) and totara (*p <* 0.001). The poorest colour match (with the highest JND values) was on totara bark (mean JND = 7.44 ± 0.249), closely followed by the lichen‐kauri substrate (mean JND = 6.79 ± 0.527). These two backgrounds were significantly different to all other substrates (all *p*‐values < 0.0001) apart from each other (*p* = 0.76). Both lichen‐kauri and titoki bark showed considerable variance and large confidence intervals (95% CI = 1.10, and 0.976 respectively). Overall, colour background matching was only ‘good’ on the kowhai substrate, with all other backgrounds presenting levels of chromatic contrast that would likely cause the moths to be easily distinguishable under the blue tit vision model.

### 3.3 Achromatic matching

**FIGURE 5:**
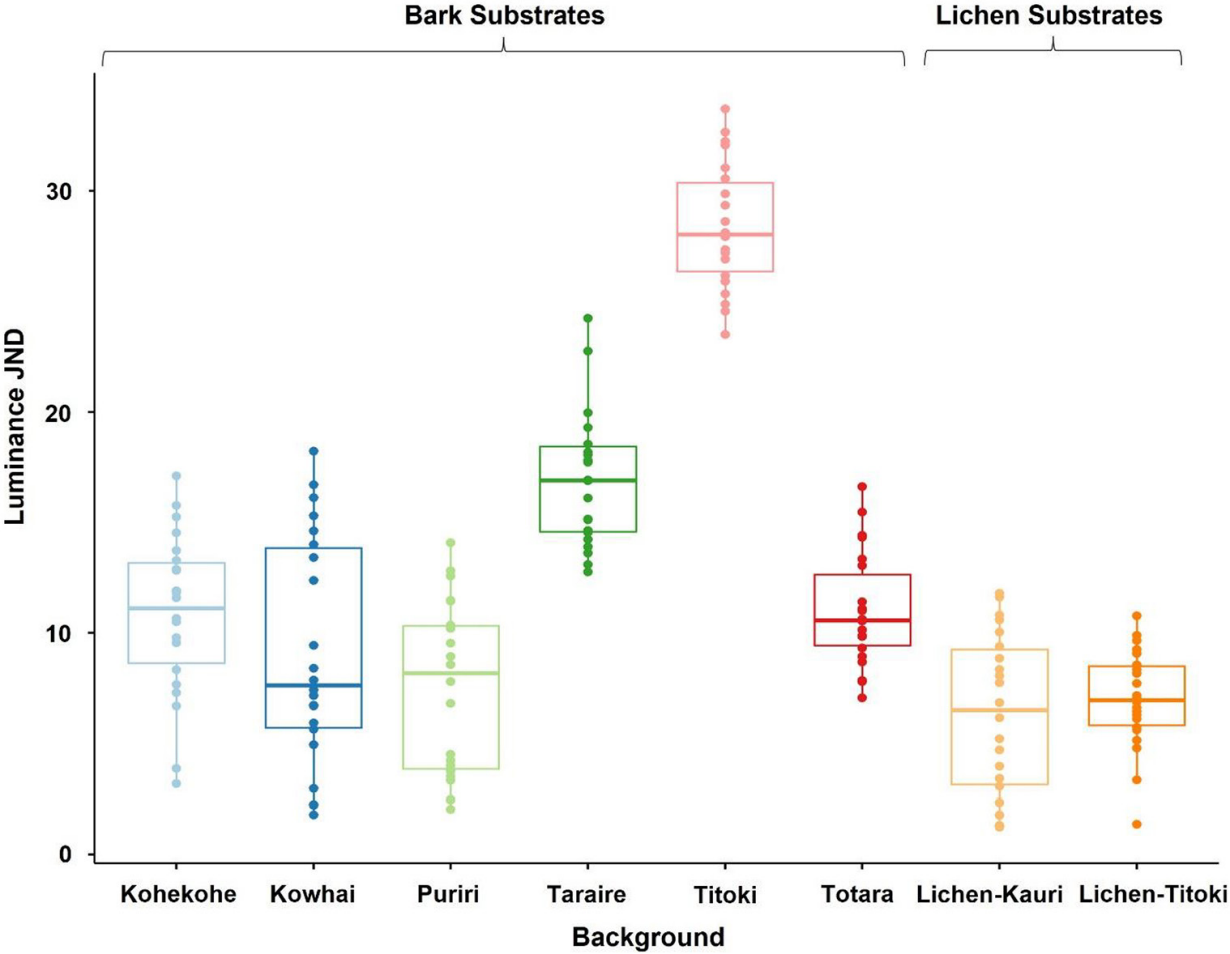
Achromatic (luminance) discrimination values (JNDs) for moths on the different background substrates as modelled under blue tit vision. All moth and background combinations presented apparently poor achromatic matches (all above 3 JND), suggesting that *Declana atronivea* would likely be distinguishable from the substrates under the current avian vision model. Moths on lichen‐kauri and lichen‐titoki had the lowest JND values (mean JND = 6.31 ± 0.77 and 6.99 ± 0.47 respectively), and titoki presented the highest JND values (mean JND = 28.3 ± 0.60). Boxplots show median values (middle line), interquartile range (box) and the range values including some outliers (dots which extend beyond the min and max of the boxplot). The horizontal grey dashed line indicates JND = 3 for comparison.

There was a significant effect of background type (*F*_7_ = 138.35, *p* < 0.001) and viewing distance (*F*_1_ = 6.86, *p* < 0.009) on luminance JND, although the interaction term between these variables was not significant (*F*_7_ = 0.036, *p* = 0.99). The variation in JND values across the background types was ineffably large (Figure 5) and none of the substrates produced a reasonable achromatic match (all above 3 JND), suggesting that based on luminance alone, the moths are likely to be distinguishable on all backgrounds. The ‘closest’ luminance matches, however, were from moths placed on lichen‐kauri (mean JND = 6.31 ± 0.766) and lichen‐titoki (mean JND = 6.99 ± 0.473). There was also found to be a small but significant effect of viewing distance on luminance JND values, irrespective of background, with mean JND values at 0.5 metres (12.7 ± 0.831) differing from that at 1 metre (11.6 ± 0.809) (*p* = 0.008; *Cohen's d* = 0.143) (Figure S2).

### 3.4 Pattern energy difference

Pattern energy difference values obtained from the granularity analysis were used to assess the degree of background pattern matching between moths and the various substrates at the different viewing distances. There were significant main effects of background type (*F*_7_ = 18.20, *p* < 0.001) and viewing distance (*F*_1_ = 254.75, *p* < 0.001) on PED, but no interaction effect between the two terms (*F*_7_ = 1.73, *p* = 0.12). This suggests that the level of background pattern matching is dependent on the substrate the moths are viewed on and the distance they are viewed from (i.e. the spatial acuity of the receiver), however, viewing distance does not influence the effect of background on PED and vice versa.

**FIGURE 6:**
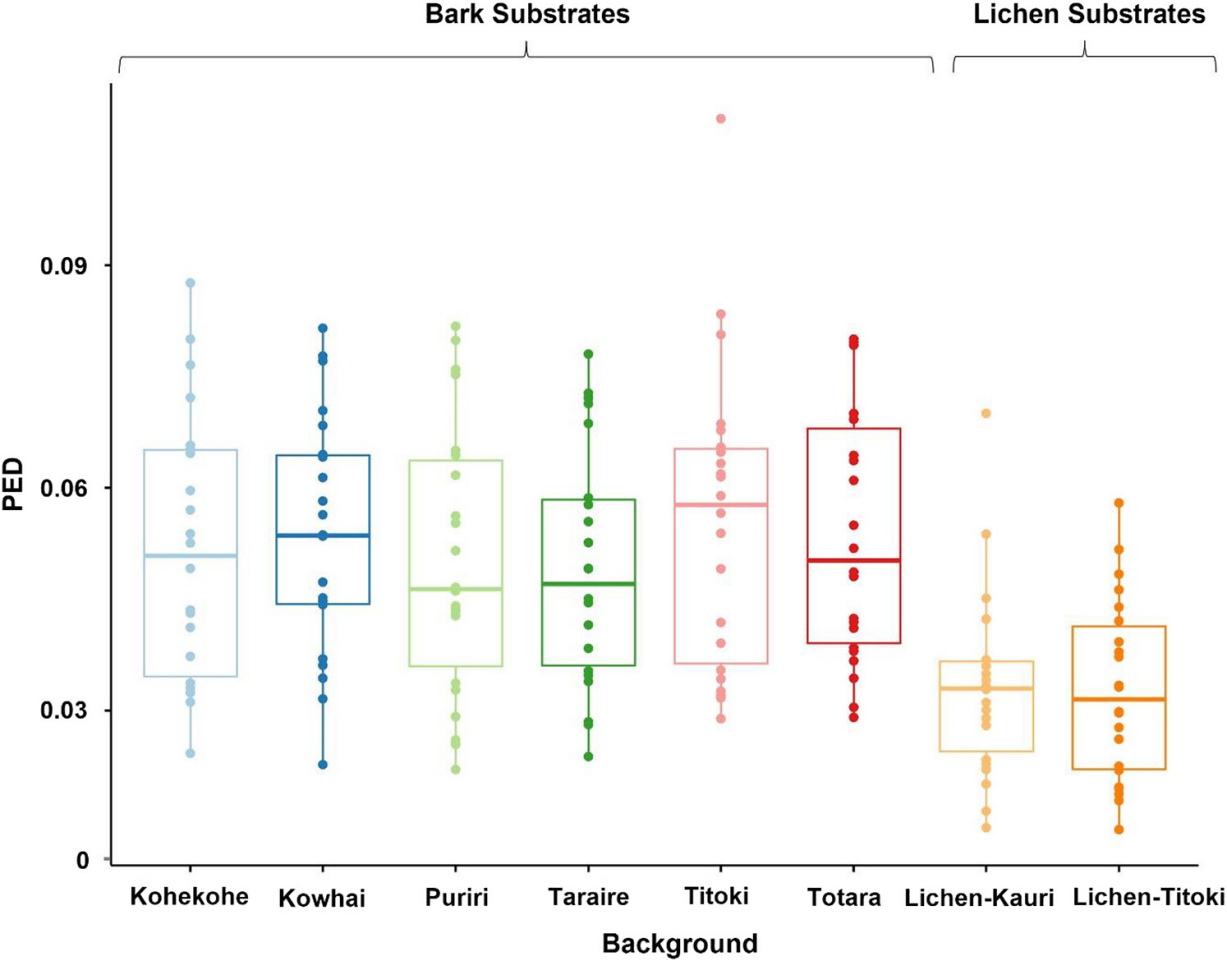
Pattern energy difference (PED) values for moths on the different background substrates. Lower values indicate a closer match and thus better background pattern matching. Here, moths had the lowest PED values on the two lichen substrates (both PED ± SE = 0.033 ± 0.003), while all of the bark substrates presented significantly higher differences in pattern energy, suggesting much better background pattern matching on lichen compared to bark backgrounds. Boxplots show median values (middle line), interquartile range (box) and the range values including some outliers (dots which extend beyond the min and max of the boxplot).

The closest pattern match to the background (with the lowest PED values) was found in moths placed on the two lichen substrates (lichen‐kauri PED = 0.033 ± 0.003, and lichen‐titoki PED = 0.033 ± 0.003; Figure 6). The PED of moths on the two lichen backgrounds did not differ from one another (*p* = 1) but were significantly different to all bark background types (all *p*‐values < 0.0001) which presented much higher PED values (taraire PED = 0.049 ± 0.003; puriri PED = 0.05 ± 0.004; kohekohe PED = 0.052 ± 0.004; kowhai PED = 0.053 ± 0.003; totara PED = 0.053 ± 0.004; titoki PED = 0.056 ± 0.004). There were no significant differences in PED values among bark backgrounds (all *p*‐values > 0.5). Overall, this indicates that *D. atronivea* moths have much greater background pattern matching to lichen substrates than to bark. Regarding viewing distance, PED values were significantly lower at 1 m (0.035 ± 0.001) compared to 0.5 m (0.059 ± 0.002) (*p* < 0.0001; Figure S3), suggesting that visual acuity may be an important factor for the degree of background pattern matching in *D. atronivea* in terms of how the patterns are perceived by avian predators.

## 4 DISCUSSION

The results from the field predation experiments show that the moth models placed on lichen substrates were attacked less often than those placed on the bark backgrounds, which is consistent with our prediction that the colour patterns of *D. atronivea* provide a survival advantage against avian predators through background matching. Interestingly, the quantification of colour and luminance contrasts was not consistent with the survival data; all of the chromatic and achromatic comparisons between *D. atronivea* moths and the different substrates had JND values that suggested an increased probability of detection from avian predators on all backgrounds, including the lichen substrates. This indicates poor background matching for colour and luminance on lichen, which contrasts with the results of the predation experiments. Conversely, the pattern contrast data show that *D. atronivea* have much lower pattern energy differences on lichen compared to the bark substrates, indicating better background matching with lichen using this camouflage metric. Considering the survival experiments and image analysis data together, the results seem to suggest that the colour and luminance of these moths may be relatively unimportant for providing protection to *D. atronivea* through background matching. Rather, it is the spatial frequency and pattern complexity of the moth forewings which provide a better match to that of the lichen substrates than to bark, thereby conferring a survival advantage. Overall, this highlights that an animal can possess relatively contrasting colour and luminance, but still gain camouflage benefits and reduced predation if they exhibit pattern elements similar to those in their environment.

*Declana atronivea* models placed on lichen had a significantly higher survival rate compared to those placed on the bark backgrounds but there was no significant survival advantage for models placed near lichen. The greater level of predation on the bark background treatment compared to on lichen backgrounds indicates that the models were more easily detected on this substrate, whereas being on lichen significantly reduced model conspicuousness to native avian predators. Given that the moth models had the exact same coloration and only differed in their background, it can be inferred that the survival advantage of the lichen treatment is due to similarity between the models and lichen substrates. These data therefore provide empirical support for the hypothesis that *D. atronivea* coloration is adapted for background matching on lichen. Similar differential survival on matching and non‐matching backgrounds has been demonstrated in the tiger beetle *Chaetodera laetescripta*. Yamamoto and Sota (2020) found that there was a strong selective advantage for artificial beetle models that were placed on colour matching substrates, whereas the models on the non‐matching backgrounds suffered significantly higher predation.

Despite the clear selective advantage of background matching demonstrated through the predation experiments, *D. atronivea* was shown to be a poor chromatic and achromatic match on both lichen and bark backgrounds under blue tit vision modelling. The assessment of these camouflage metrics was thus contrary to our prediction that colour and luminance contrasts should be lowest on lichen to reflect the adaptation for background matching. Stobbe et al. (2009) found that both achromatic and chromatic cues were important to blue tits when visually searching for prey, and the chromatic differences between prey and background were particularly important for increasing predation risk. The apparent high saliency of the chromatic and achromatic properties of *D. atronivea* compared with the bark and lichen backgrounds should therefore make the moths equally conspicuous to predators on both substrates, yet models placed on lichen had significantly higher survival during the predation experiments. This seems to suggest that colour and luminance do not accurately predict survival through background matching in this species.

Numerous studies have shown that background matching may not need to be as accurate to confer a survival advantage, particularly when on a visually complex substrate (e.g. Dimitrova & Merilaita, 2010, 2012; Hughes et al., 2019; Murali et al., 2021; Rowe et al., 2021; Xiao & Cuthill, 2016). This is because a higher amount of visual information present on a substrate can interfere with predator perceptual processes (Merilaita et al., 1999). A more complex background composed of many contrasting chromatic and achromatic elements, as well as different shapes and spatial frequencies, can increase the noise of the visual scene through introducing ‘visual clutter’ (Dimitrova & Merilaita, 2010; Merilaita et al., 2017; Xiao & Cuthill, 2016; Rowe et al., 2021). Visually searching for prey is a very demanding perceptual task for predators and the addition of such feature congestion from the background can reduce the signal‐to‐noise ratio, thereby increasing the amount of information that needs to be processed and potentially relaxing the requirement for more precise background matching (Dimitrova & Merilaita, 2010, 2012; Merilaita, 2003). It is possible that the visual complexity of the lichen backgrounds mitigates the poor levels of chromatic and achromatic matching in *D. atronivea*, allowing the moths to have greater survival on these substrates compared to the seemingly more homogeneous bark backgrounds, as demonstrated in the predation experiments.

In contrast to the colour and luminance metrics, background pattern matching appears to be a much better predictor of moth survival. The granularity analysis presented pattern energy differences between the moths and the backgrounds that were significantly lower on the lichen substrates compared to bark, indicating a greater level of camouflage on lichen, which corresponds with the field survival data. As previously mentioned, lichen likely presents a more visually complex background than plain bark substrates and as such, is a better match to the intricate wing pattern elements of *D. atronivea*. The combined spatial information of the moth on a lichen background could act to reduce the signal‐to‐noise ratio, making it more difficult for predators to visually segment the two stimuli. The importance of pattern elements for background matching can also be seen in studies which have investigated background choice and body orientation to optimise concealment by moths, which have shown that some moths orient their bodies to better match the pattern elements of bark backgrounds and thus maximise their concealment (Kang et al., 2012; Webster et al., 2009). Pattern matching has similarly been shown to be a significant predictor of survival in ground‐nesting birds (Troscianko et al., 2016).

Pattern perception in birds is thought to be mediated by the double cones, so the pattern energy difference in this study was quantified using the luminance channel of the blue tit vision model. In this way, pattern and luminance perception of *D. atronivea* coloration by avian predators are likely to be connected, which presents a conundrum given that the moths displayed a close pattern match with lichen but were achromatically divergent. Additionally, both luminance JND and pattern energy difference were influenced by viewing distance, with overall contrasts significantly lower at 1 m compared to 0.5 m. This suggests that visual acuity is important for the perception of these visual cues. Luminance is thought to mediate long‐range detection (Osorio, Miklósi, et al., 1999; Osorio & Vorobyev, 2005; Osorio, Vorobyev, et al., 1999), so perhaps at greater distances the moths would appear a better achromatic match to the lichen backgrounds. Another possible explanation for this deviation between luminance and pattern perception is that the colour patterns of *D. atronivea* may actually function to reduce predation via a different camouflage mechanism rather than background matching. The combination of pattern similarity, high contrast markings, and the fact the *D. atronivea* did not match the chromatic or achromatic properties of either lichen or bark backgrounds, yet still had a survival advantage on lichen, suggests this species may instead (or perhaps additionally) be adopting disruptive coloration as a camouflage strategy (Merilaita & Lind, 2005; Robledo‐Ospina et al., 2017). Disruptive coloration comprises of typically high contrast markings which create false edges and break up surface continuity, thereby hindering detection or recognition of animal's true form. It has been shown to function independently of background matching (Schaefer & Stobbe, 2006; Stevens et al., 2006), so the selection pressure for visual similarity with the substrate may be reduced, which may explain the lack of chromatic and achromatic coherence with the lichen backgrounds. Investigation into the contribution of disruptive coloration as a camouflage mechanism in *D. atronivea* is an avenue for future work.

## 5 CONCLUSIONS

To summarise, *D. atronivea* coloration confers a significant survival advantage against native avian predators when on lichen backgrounds compared to plain bark. This suggests that *D. atronivea* coloration is likely adapted for background matching on lichen substrates. The lack of consistency between the survival data and the chromatic and achromatic contrasts on lichen suggests that colour and luminance alone do not predict background matching well in this species. Instead, patterns of predation were supported by granularity analysis which showed the lowest pattern energy differences between moths and lichen substrates, indicating that the pattern elements of *D. atronivea* are most important, and likely to be driving the benefits of background matching for this species. This study is one of the first to integrate vision modelling, quantitative image analysis and field predation experiments using realistic models to objectively quantify the level and functional significance of background matching, and the first integrated approach to demonstrate the importance of pattern similarity in reducing predation, in the absence of matching colour and luminance.

## AUTHOR CONTRIBUTIONS

All authors conceived the ideas and designed the methodology of this study. Cassandra Mark collected and analysed the data and led the writing of the manuscript as based on their PhD thesis. All authors contributed critically to the drafts and gave final approval for publication.

## CONFLICT OF INTEREST

The authors have no relevant financial or non‐financial conflict of interest to disclose.

## Supporting information


Appendix S1

## Data Availability

Data available from Figshare https://doi.org/10.6084/m9.figshare.18316529.v1 (Mark et al., 2022).
