# Supplementary material for: Camouflage in lichen moths: Field predation experiments and avian vision modelling demonstrate the importance of wing pattern elements and background for survival
Source: J Anim Ecol. 2022 Oct 8;91(12):2358–69. doi: 10.1111/1365-2656.13817 (PMC10092008; doi:10.1111/1365-2656.13817)
Supplement: Supplementary file 1 — Appendix S1 [file JANE-91-2358-s001.docx]

Supplementary Material

**Title:** Camouflage in Lichen Moths: Field Predation Experiments and Avian Vision Modelling Demonstrate the Importance of Wing Pattern Elements and Background for Survival.

**Authors:** Mark, C.J^a*^., O’Hanlon, J.C^b^., & Holwell, G.I^a^.

^a^School of Biological Sciences, The University of Auckland, Building 110, 3A Symonds Street, Auckland 1010, New Zealand

^b^School of Science and Technology, University of New England, Armidale, NSW 2351, Australia

| **Contents:** | Page |
| --- | --- |
| **Supplementary Methodology for:** |  |
| Developing the Artificial Models for Field Predation Experiments | 2 |
| Image Acquisition for Image Analysis | 3 |
| Image Processing and Vision Modelling | 5 |
| **Supplementary Figures:** |  |
| Figure S1 | 7 |
| Figures S2 | 8 |
| Figure S3 | 9 |
| **Supplementary References** | 9 |

**Developing the Artificial Models for Field Predation Experiments:**

The models were produced from a photograph of a single fresh, intact specimen using Adobe Photoshop (ver. 22.4.2). As *D. atronivea* can be highly variable in their wing colour pattern, geometric-morphometric analysis was first used to determine which specimen best represented the ‘average’ of the population. This allowed us to control for this variation while keeping the colour patterns within a natural range. To ensure that the artificial models were appropriately calibrated to bird vision and reflected as close to real *D. atronivea* as possible (Merrill et al., 2012), we first used spectrophotometry to quantify the wing colouration of the moths. Spectral reflectance values of *D. atronivea* wing colour patterns were measured using the Ocean Optics USB2000+ spectrometer with a PX2 (Pulsed Xenon Lamp with a wavelength range of 220-750 nm) light source fitted with a Lab-grade Reflection Probe (numerical aperture of 0.22 ± 0.02; Ocean Optics, FL). The probe was fitted into a RPH Reflection Probe Holder (Ocean Optics, FL) such that illumination and detection of reflected light were performed 45 degrees to the surface of the wing. A white Spectralon standard (99% reflectance; Labsphere) was used to calibrate the spectrometer. Spectral measurements were taken from three randomly positioned points per colour patch (white, black, and grey-brown) on the forewings of five male and five female *D. atronivea* moths. From these, an average reflectance was obtained which was used to generate reflectance curves for each colour. Preliminary analysis of the reflectance curves revealed that the white wing scales of *D. atronivea* reflect in the UV part of the spectrum. To account for this, we removed the white pixels from the models using Adobe Photoshop (ver. 22.4.2) to render the white sections transparent so that only grey-brown and black colour patches were printed directly onto white UV reflecting paper. To ensure the spectral output of the printing paper was matched closely to that of the white wing scales, we sampled reflectance measurements across a range of paper brands. Spectra were taken from three randomly positioned points on a representative piece of each paper brand and the average was used to generate reflectance curves for each type. Measurements were also taken after applying a layer of Plasti-Kote Clear Acrylic spray paint. Acrylic spray is often used to make artificial models waterproof (Finkbeiner et al., 2012; Walton, & Stevens 2018), and it did not affect the spectral profile of the papers.

The spectral data were analysed in the ‘R’ package, ‘PAVO’ (Maia et al., 2019), allowing us to compare the reflectance curves of the different paper types to that of the white wing scales. Colour distances and just noticeable differences (JNDs) (as modelled through blue tit *Cyanistes caeruleus* vision) were also calculated for these comparisons. From these analyses, Whatman Filter Paper was confirmed to be the closest match to the white wing patches being within one JND and thus not discernible by avian vision as being different to the actual moths. This corresponded to what has been found in other studies (Finkbeiner et al., 2012; Walton & Stevens, 2018). The ink for the black and grey-brown wing colour patches were calibrated in an iterative process by printing the models onto the Whatman filter paper using different tones of the coloured inks and measuring and comparing the spectra as described above.

**Image Acquisition for Image Analysis:**

Digital photographs of *D. atronivea* moths were taken on the bark of native trees (kohekohe *Dysoxylum spectabile*; kowhai *Sophora microphylla*; puriri *Vitex lucens*; taraire *Beilschmiedia taraire*; titoki *Alectryon excelsus*; totara *Podocarpus totara*) and on lichen substrates that were found on kauri (“kauri-lichen”, which primarily consisted of a mixture of *Parmotrema, Hypotrachyna, Heterodermia* lichen species) and titoki (“titoki-lichen”, including lichen species of the *Parmelia*, *Flavoparmelia*, and *Punctelia* genera) located in the grounds of the University of Auckland (between 36°50'59.0"S 174°46'10.2"E and 36°51'00.0"S, 174°46'16.0"E) during January 2020. These substrates were selected because they were found to be common in the forests of Oratia and Matuku reserves where the field-predation experiments took place. The species variability of the lichen substrates also reflects the natural assemblages found in those forests. Photographs were captured during the day and under cloudy conditions to ensure more even and diffuse lighting.

To avoid the risk of moths moving or flying away during photography, we used dead specimens (n = 11) that had previously been dried out into a natural resting position. The moths were mounted onto pins by gluing the underside of the body to a small square of cardboard which was then glued to the flat head of a sturdy dressmaker pin, and pinned to the substrate. All moth specimens were photographed on the same individual tree of each tree species. Care was taken to ensure the specimens were all pinned at the same orientation (0 ° of the vertical plane), though the position of the specimens on each substrate was randomised in terms of height and placement around the tree trunk so that the images did not represent the same patch of background. All photos were taken at a distance of 0.5 metres from the substrate. A white Spectralon standard (99% reflectance; Labsphere) attached to a scale bar was placed at the side of each photo.

Acquisition of digital images was carried out following the protocols described in Troscianko & Stevens (2015) and the corresponding Image Calibration and Analysis Toolbox user guide. Photographs were taken on a Sony A7 DSLR fitted with a Nikon El-Nikkor 80 mm enlarging lens and attached to a tripod. The camera was modified through quartz conversion to allow for full-spectrum sensitivity (Nikon on Broadway, NSW, Australia), including UV wavelengths important for avian vision. The spectral sensitivities of the camera are as follows: UV: 340–400 nm (peak 380 nm), SW: 410–550 nm (peak 460 nm), MW: 420–630 nm (peak 540 nm), LW: 570– 690 nm (peak 650 nm). Two sets of images, photographed in RAW format, were taken for every specimen and background combination: one using a visible pass filter (Baader UV/IR Cut filter) which blocks UV and infrared wavelengths allowing for only human-visible images, and another using a UV pass filter (Baader Venus U filter) which allows only the transmission of UV wavelengths (300-400 nm) for the acquisition of UV images. Xume magnetic lens adapters were attached to the camera lens and the two filters to allow for easier transition between the filters during photography. Exposure bracketing was used to obtain the best exposure for the images.

**Image Processing and Vision Modelling:**

Visual modelling and analysis of the images were conducted using the open-source Multispectral Image Calibration and Analysis (MICA) toolbox (Troscianko & Stevens, 2015) and the integrated Quantitative Colour Pattern Analysis (QCPA) Framework (van den Berg et al., 2020) for ImageJ (version 1.5.3; Schneider et al., 2012). Images were first screened to check for overexposure and then a RAW image in both the visible and UV spectrum for each specimen and background combination were selected for the following image processing and analyses. Each pair of photographs were then converted into a multispectral image, which consists of a stack of images captured at different wavelengths (Troscianko & Stevens, 2015). This generated five image channels corresponding to long wavelength (LW), medium wavelength (MW), and short wavelength (SW) (i.e., the visible red (vR), green (vG), and (vB) blue acquired through the visible pass filter), and UV (ultraviolet red (uR) and ultraviolet blue (uB) from the UV pass filter). Regions of interest (ROIs) were then selected for measurement, using the polygon tool to carefully draw around the outline of the *moth* ROI and then the rectangle tool to specify the *background* ROI. As the moth was centred in the image it was initially encompassed within the background region, so the XOR function was used to exclude the moth from the background ROI.

To objectively assess the colour pattern properties of *D. atronivea*, each multispectral image was converted to animal-vision cone-catch quanta. Cone-catch images are based on the spectral sensitivities and photoreceptor responses of a particular visual system. As *D. atronivea* reflect in the UV, it was important to model a receiver with the correct spectral sensitivities. Unfortunately, there are currently no complete vision models for native New Zealand passerines, and the retinal properties of relevant avian predators of *D. atronivea*, such as tui *P.* *novaeseelandiae* and pīwakawaka *R. fuliginosa*, are unknown. However, photoreceptors and spectral sensitivities of birds tend to be phylogenetically conserved (Hart, 2001), so here we modelled the visual system of the blue tit *C. caeruleus* as a proxy for native avian predators.


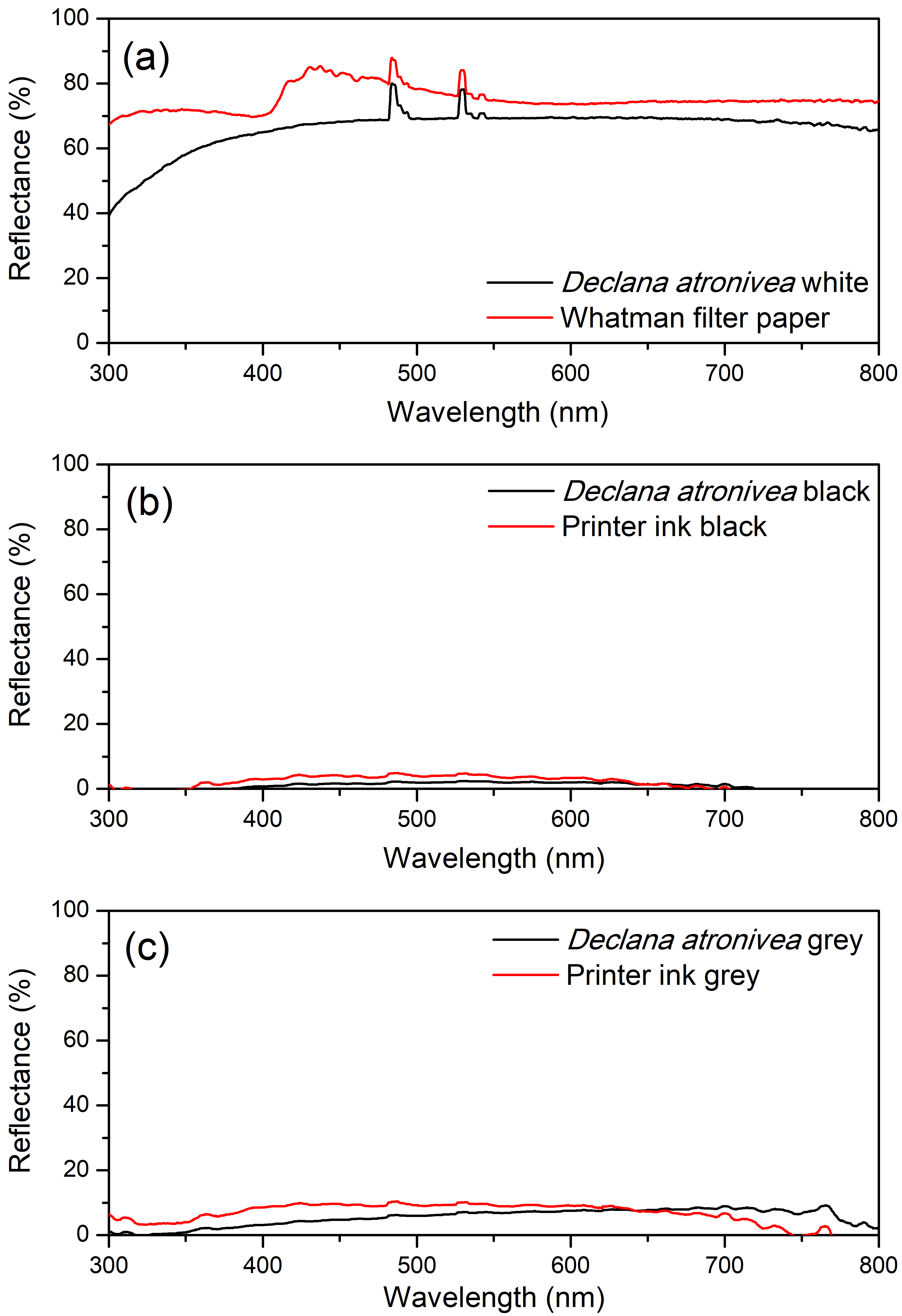


**Figure S1**. Representative UV-Vis reflectance spectra of D. atronivea forewings colours compared with calibrated printing paper and ink used for creating paper moth models: a) D. atronivea white wing patches and Whatman filter paper; b) D. atronivea black wing patches and the calibrated black printer ink; c) D. atronivea grey/brown wing scales and the calibrated grey/brown printer ink.


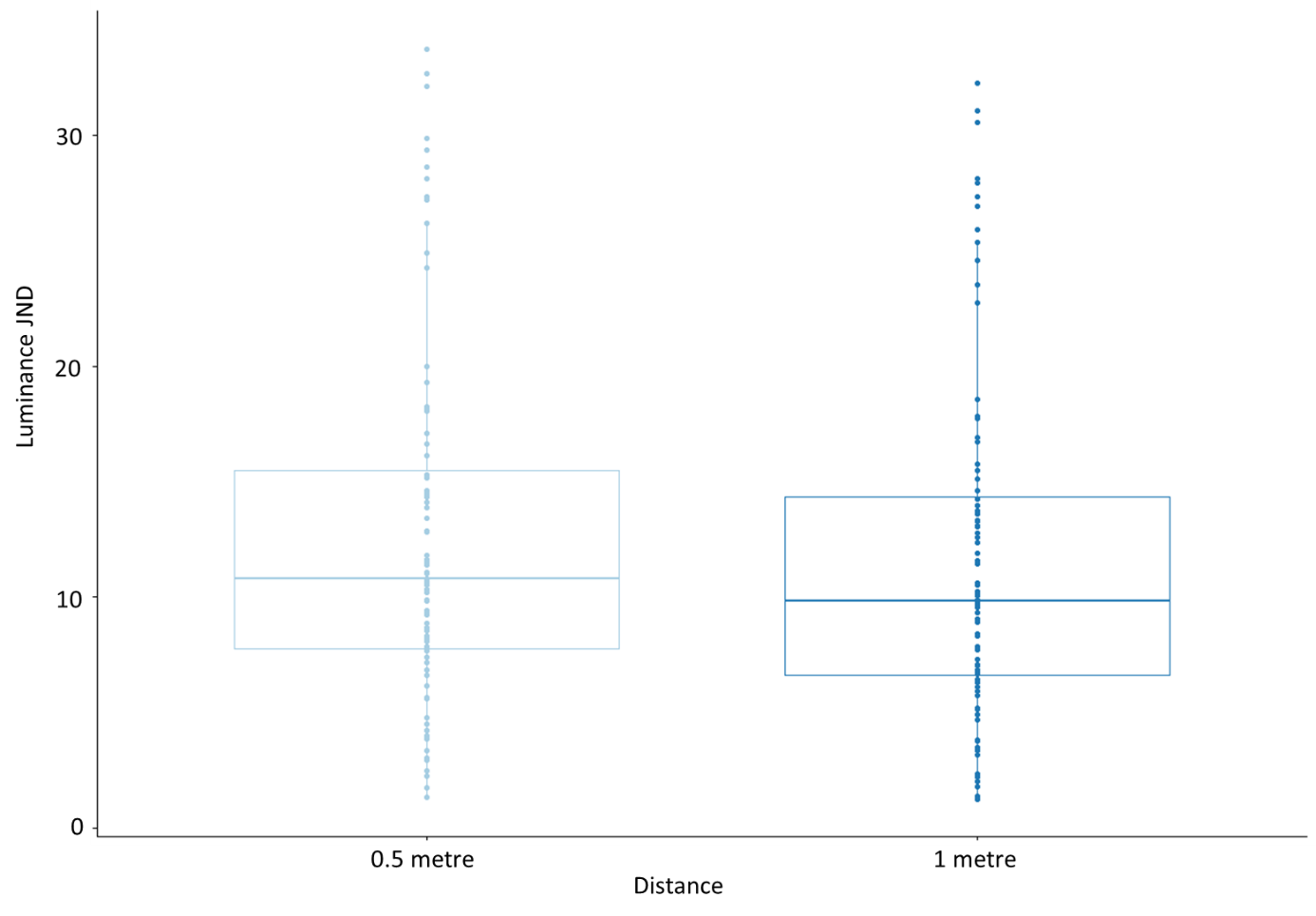


**Figure S2.** Effect of distance on luminance JND values. Luminance JND values at 0.5 metre (12.7 ± 0.831) and 1 metre (11.6 ± 0.809) viewing distances were significantly different, suggesting an effect of spatial acuity on luminance perception. Boxplots show median values (middle line), interquartile range (box), and the range values including some outliers (dots).


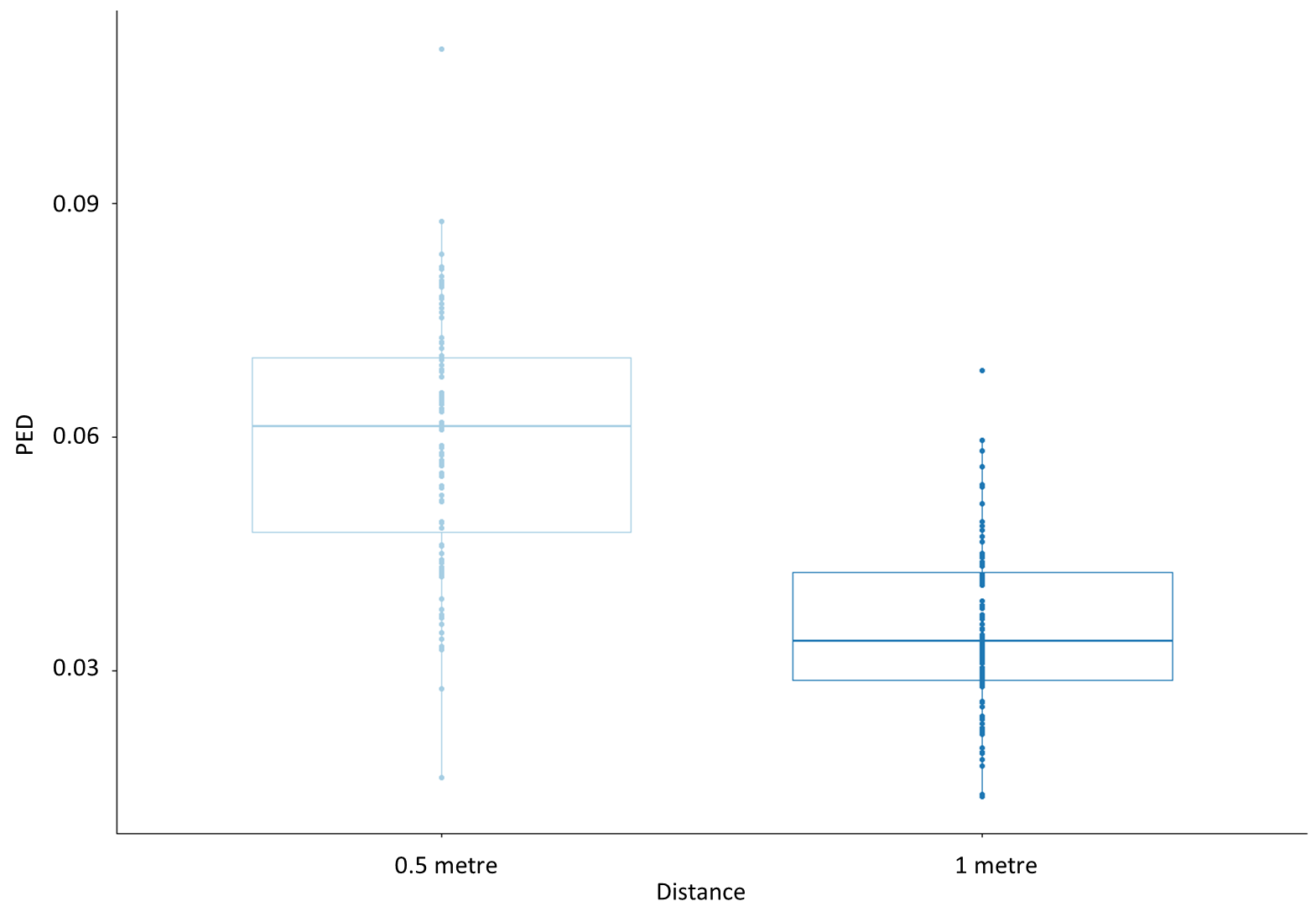


**Figure S3**. Effect of distance on PED values. PED values were significantly higher at 0.5 metre (0.059 ± 0.002) than 1 metre (0.035 ± 0.001) viewing distances were significantly different, suggesting a possible effect of spatial acuity on pattern perception. Boxplots show median values (middle line), interquartile range (box), and the range values including some outliers (dots).

**References:**

Finkbeiner, S. D., Briscoe, A. D., & Reed, R. D. (2012). The benefit of being a social butterfly: communal roosting deters predation. *Proceedings of the Royal Society B: Biological Sciences*, *279*(1739), 2769-2776.

Hart, N. S. (2001). The visual ecology of avian photoreceptors. *Progress in retinal and eye research*, *20*(5), 675-703.

Maia, R., Gruson, H., Endler, J. A., & White, T. E. (2019). pavo 2: new tools for the spectral and spatial analysis of colour in R. *Methods in Ecology and Evolution*, *10*(7), 1097-1107.

Merrill, R. M., Wallbank, R. W., Bull, V., Salazar, P. C., Mallet, J., Stevens, M., & Jiggins, C. D. (2012). Disruptive ecological selection on a mating cue. *Proceedings of the Royal Society B: Biological Sciences, 279*(1749), 4907-4913.

Schneider, C. A., Rasband, W. S., & Eliceiri, K. W. (2012). NIH Image to ImageJ: 25 years of image analysis. *Nature methods*, *9*(7), 671-675.

Troscianko, J., & Stevens, M. (2015). Image calibration and analysis toolbox–a free software suite for objectively measuring reflectance, colour and pattern. *Methods in Ecology and Evolution*, *6*(11), 1320-1331.

van den Berg, C. P., Troscianko, J., Endler, J. A., Marshall, N. J., & Cheney, K. L. (2020). Quantitative colour pattern analysis (QCPA): a comprehensive framework for the analysis of colour patterns in nature. *Methods in Ecology and Evolution, 11*(2), 316-332.
